# The Adulteration of Drugs

**Published:** 1899-10-21

**Authors:** 


### THE ADULTERATION OF DRUGS.

The report of the Principal Chemist of the Government Laboratories, which has recently been published, is an interesting document, seeing that these laboratories have of necessity to act as the final court of appeal in various questions concerning analysis, as, for example, in regard to all disputed analyses under the Food and Drugs Act. Bearing in mind the very large number of samples which are annually taken by the sanitary authorities for examination under this Act, we venture, however to think that the strikingly little work done by the Government Laboratories in the analysis of drugs is indicative of want of energy on the part of the sanitary authorities in putting into operation that side of the Food and Drugs Act which applies to these compounds. There can, indeed, be no doubt that a lamentable degree of laxity prevails in this matter, and that the number of samples of drugs examined by the authorities is ridiculously small when compared with those of food seized for the same purpose. As illustrating the freedom and impunity with which the substances dealt in by chemists are apt to be adulterated, we may refer to an investigation which we lately made in regard to the composition of belladonna plasters. Our attention had been drawn to the fact that the amount of belladonna contained in certain much advertised belladonna plasters was far below that which is ordered by the Pharmacopœia, and to ascertain what was the exact position of affairs we had samples taken from a considerable number of chemists' shops, including those of some makers who rather posed before the public as making a speciality of these plasters. The result was amusing, although somewhat destructive of one's faith in man, and especially in chemists. Unfortunately it was impossible to publish the report as it was received from our chemist, containing as it did the names of all the parties implicated. We had no desire to be involved in a series of actions for libel, and so the matter was allowed to drop.

The search for adulteration should not be limited to questions of butter, and pepper, and coffee, and such like articles of diet. Drugs, on the purity of which we trust in the treatment of disease, should be subjected to just as strict analysis as the foods we eat. Notwithstanding the greatly widened field from which the physician of to-day draws his means of treating disease compared with the shelf-ful of bottles which constituted the therapeutic armamentarium of the apothecary of days gone by, the fact remains that drugs form a large, and in many cases the principal means on which we have to depend in the control of those ailments by which our patients are affected. Thus it happens that the honesty of the druggist is the very pivot on which hangs the life of the patient in many cases. How frequently this is the case is a matter of common experience. So strongly has this been felt and recognised that in the Sale of Food and Drugs Act, 1875, heavy penalties were imposed on all who sell to the prejudice of the purchaser any drug which is not of the nature, quality, and substance asked for. As is well known, there has been plenty of litigation on the subject, and in regard to food many adulterators have escaped in consequence of a somewhat liberal reading of certain clauses in the Act, which provide that no offence shall be deemed to have been committed where any matter or ingredient not injurious to health has been added to the food or drug because the same is required for the preparation or production thereof as an article in a fit state for carriage or consumption, or where the food is unavoidably mixed with any extraneous matter in the process of collection or preparation. But in regard to such drugs as are included in the British Pharmacopœia all doubt and difficulty is removed. By the Medical Act of 1858 it is enacted that "the General Medical Council shall cause to be published under their direction a book containing a list of medicines and compounds, and the manner of preparing them, together with the true weights and measures by which they are to be prepared and mixed." This book is "The British Pharmacopœia," which gives the definition, and fixes the legal standard of all the drugs and preparations named therein, so that in regard to such things there need never be any doubt as to what is really meant by a drug being of the "nature, quality, and substance asked for." It must conform with the characteristics given in the Pharmacopœia, or it must be wrong.

Under these circumstances we decided to ascertain how far druggists were in the habit of complying with the directions of the Pharmacopœia in regard to some of the more ordinary preparations commonly sold over the counter. Now, belladonna plasters, as well as being prescribed by physicians, are also commonly purchased by the public even without a prescription, and it appeared to us that such a preparation would be a good one on which to test the *bonâ fides* of the manufacturing druggist and his readiness to supply the public with reliable goods prepared according to the legal standard. With this object in view we purchased samples of belladonna plaster from 16 well-known firms or their agents, and requested Dr. B. H. Paul, of 13, Fenchurch Avenue, to undertake their analysis, so as to ascertain how far they complied with the conditions specified in the British Pharmacopœia.

We certainly were not prepared for the result, nor do we think that any of our readers, however much some of them may be ready to doubt the honesty of man, will be quite prepared for the extraordinary amount of adulteration shown in the subjoined list. It must be premised that, according to the British Pharmacopœia, belladonna plaster "contains 0·5 per cent. of the alkaloids of belladonna root."

The following is Dr. Paul's report, from which, however, in deference to the law of libel, we have omitted the names of all firms except those whose plasters were of full strength:—

"Sirs,—In accordance with your instructions I have submitted to analysis the samples of belladonna plaisters marked as under, and supplied by you on December 22nd and on subsequent dates, with the following results—

| | Alkaloid p.c. |
|---|---|
| Seabury and Johnson ... ... ... ... | 0·52 |
| Name withheld ... ... ... ... ... | 0·06 |
| " " ... ... ... ... ... | 0·04 |
| " " ... ... ... ... ... | 0·19 |
| " " ... ... ... ... ... | 0·16 |
| " " ... ... ... ... ... | 0·16 |
| " " ... ... ... ... ... | 0·12 |
| " " ... ... ... ... ... | 0·12 |
| " " ... ... ... ... ... | 0·13 |
| " " ... ... ... ... ... | 0·27 |
| " " ... ... ... ... ... | 0·11 |
| " " ... ... ... ... ... | 0·13 |
| May's Drug Stores ... ... ... ... | 0·56 |
| Name withheld ... ... ... ... ... | 0·09 |
| " " ... ... ... ... ... | 0·12 |
| " " ... ... ... ... ... | 0·27 |

Benj. H. Paul.

Analytical Laboratory, 13, Fenchurch Avenue, E.C., January 26th, 1899."

We hardly need to comment upon the facts here displayed. The list tells its own tale and points its own moral, for we find opposite the names of firms well known, and, indeed, well advertised as makers of these things, figures showing that their plasters contain but a small quantity of their proper ingredients—the proportion of the alkaloids going down and down, till in one case the amount is less than one-twelfth of what the law requires.

Clearly, however, it is possible to obtain a belladonna plaster of the full strength, and there, no doubt, are many chemists where such a plaster can be bought. We should be the last to suggest that such adulteration as is here displayed is universal among chemists and druggists; but what we do say is that to those who act honourably in the matter a great wrong is done. The wrong done to the public by such adulteration is obvious enough, for it evidently is possible to enter a good shop and to buy a pharmacopœial preparation stamped with the name of a reputedly good manufacturing druggist, and yet to be supplied with an article which is ludicrously below the standard strength. But to the honest druggist also the hardship is very great in that he has to struggle against an unscrupulous competition, and that no one believes him however much he may declare that his drugs are pure. The public are helpless in the matter, and it is only by the action of the inspectors under the Food and Drugs Act, or by the aid of such an exposure as that which we make to-day that such unprincipled competition can be kept in check.

Unfortunately, the matter does not end with belladonna plasters. We are assured that much the same sort of sophistication goes on in many branches of the drug trade, and that, trusting to the absolute helplessness of the purchaser, druggists only too often supply drugs which are anything but of the "nature, quality, and substance asked for."

